# A new species of *Labiobaetis*Novikova & Kluge, 1987 (Ephemeroptera: Baetidae) from the southern Western Ghats in India, with comments on the taxonomic status of *Labiobaetis*

**DOI:** 10.1093/jis/14.1.86

**Published:** 2014-07-07

**Authors:** T. Kubendran, T. Rathinakumar, C. Balasubramanian, C. Selvakumar, K. G. Sivaramakrishnan

**Affiliations:** 1 Department of Zoology, Thiagarajar College (Autonomous), Madurai-625 009, Tamilnadu, India; 2 Department of Zoology, University of Madras, Guindy Campus, Chennai-600 025, India; 3 Department of Zoology, Madras Christian College (Autonomous), Tambaram East, Chennai-600 059, India

**Keywords:** mayfly, systematics, distribution, southern India

## Abstract

A new species of *Labiobaetis*Novikova & Kluge, 1987 , *Labiobaetis soldani* sp. nov., is described from the larvae and reared male and female imagoes from Gadana River in the southern Western Ghats in India. Brief ecological notes are appended. The taxonomic status of *Labiobaetis* is commented on in light of the morphological traits of the larvae and associated imagoes.

## Introduction

There is considerable scarcity in the taxonomic studies on Oriental Baetidae, particularly in India, which potentially harbors a rich diversity of baetine mayflies ( Soldan 2001 ; Gattolliat and Nieto 2009 ). However, generic limits of Baetidae are being revised periodically at the global level through simultaneous analysis of larval and imaginal traits and by establishing new genera and synonymizing several previously established genera in this family. The genus *Labiobaetis*Novikova & Kluge, 1987 is not an exception to this. For instance, Navas (1931) and Gillies (1949) described *Pseudocloeon rubellum* and *Baetis palmyrae* , respectively, from the state of Maharashtra of India. Those species were subsequently transferred to the genus *Labiobaetis* by McCafferty and Waltz (1995) . Species of Baetidae described in several earlier publications ( Kapur and Kripalani 1963 ; Dubey 1970 , 1971) have not been reexamined in the context of the current generic concept of *Labiobaetis* , because descriptions of the crucial larval stage are unavailable. However, around 15 nominal species, mostly belonging to *molawinensis* species group of the Oriental region of *Baetis* , were transferred to *Labiobaetis* ( McCafferty and Waltz 1995 ; Sivaramakrishnan et al. 2009 ).

As part of a continued effort to explore the Ephemeroptera fauna of the streams of the southern Western Ghats, a new species of the genus *Labiobaetis* was collected as larvae reared to the respective imago. This is the first record of this genus in peninsular India, extending its distributional range from Sri Lanka northwards to southern peninsular India. Larval and imaginal descriptions and differential diagnoses are provided. Observations on the taxonomic status of this enigmatic genus are also appended in light of the discovery of this new species.

### Generic diagnoses

*Labiobaetis* spp. can be diagnosed in the larval stage by the following combination of characters: 1) distolateral notch on the antennal scape present Figure 5 ); 2) right prostheca maniform ( Figure 11 ); 3) presence of a distomedial concavity on segment 2 of maxillary palps ( Figure 12 ); 4) width of the paraglossa of labium more than 1.8 times wider than the glossa ( Figure 13 ); 5) apex of labial palp slightly pointed ; 6) second segment of labial palp with a broad thumblike distomedial projection laterally rounded; 7) femoral villopore absent ( Figure 6 ); 8) projection at inner distal end of paraproct absent ( Figure 14 ); and 9) patch of notched scale of paraproct present.

**Figure 1–7.**
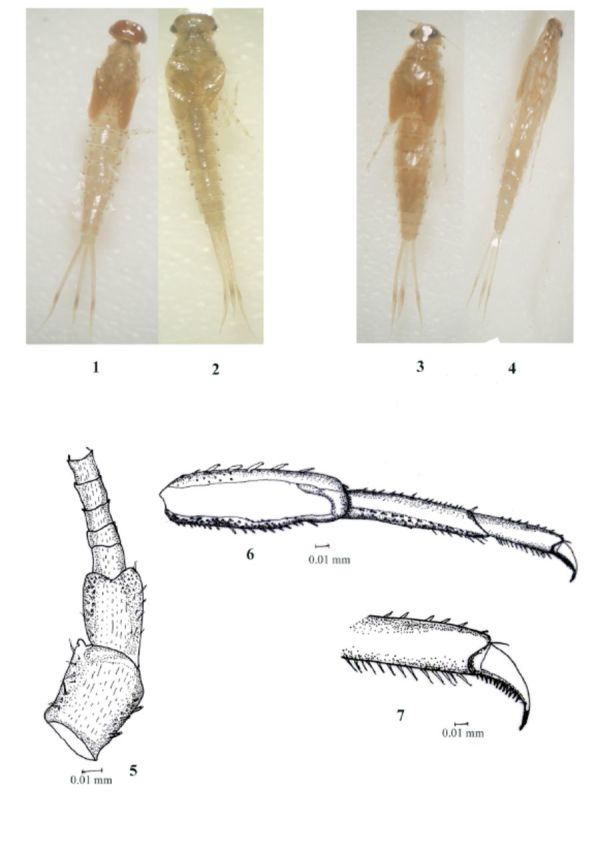
Labiobaetis sp.nov., 1, male dorsal view; 2, Male ventral view; 3, Female dorsal view; 4, Female ventral view; 5, Antennae; 6, Leg; 7, Claw

The genus *Labiobaetis* can be diagnosed in the imaginal stage by the following combination of characters: 1) anterior margin of frons with medial ridge straight in lateral view, 2) apical segment of forceps globular ( Figure 22 ), and 3) forewings with double intercalary vein.

**Figure 8–14.**
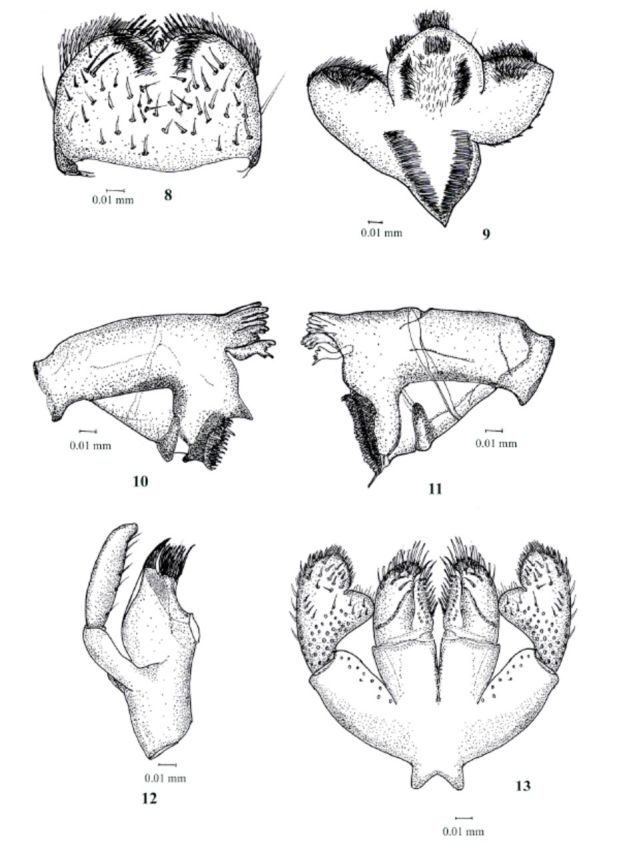
Labiobaetis soldani sp.nov., 8, Labrum; 9, Hypopharynx; 10, Left mandible; 11, Right mandible; 12, Left maxillae; 13, Labium

### Nomenclature

This publication and the nomenclature it contains have been registered in ZooBank. The LSID number is:

urn:lsid:zoobank.org :pub:A4273A5C-6CB9-415B-97F6-706BC85CD1B0

*Labiobaetis soldani* **sp. nov. (Figures 1–22)**

### Larva

Maximal length, fully grown male ( Figures 1 and 2 ); body 4.0 mm; cerci 2.0 mm; terminal filament 1.5 mm; fully grown female ( Figures 3 and 4); body 4.8 mm; cerci 2.0 mm; terminal filament 1.7 mm.

**Figure 14–22.**
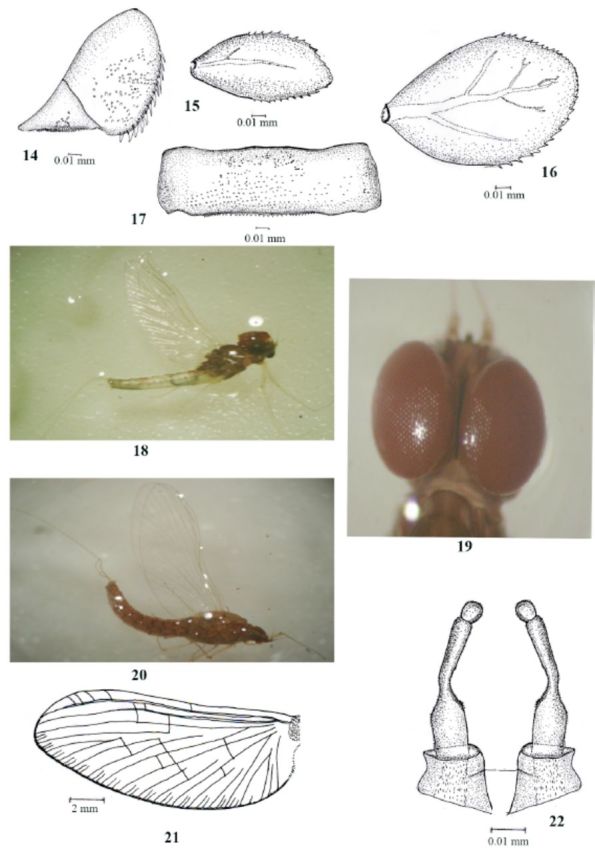
Labiobaetis soldani sp.nov., 14, Paraproct; 15, Gill I; 16, Gill IV; 17, Abdominal terga; 18, Adult male; 19, Male head; 20, Adult female; 21, Fore wing; 22, Genitalia

Head: coloration almost uniformly light brown, antennae light yellow (Figure 5); scapes with notch at distolateral margin and few short fine setae present on the surface. Labrum ( Figure 8 ): rounded, with an arc and stout setae, long and thin setae medially; distal margin bordered with setae, distolaterally 14 feathered bristles; ventrally with 4 stout, 2 long setae and distomedial arc of very thin setae. Left mandible ( Figure 10 ): stout prostheca apically with denticules and a comb-shape structure; margin between prostheca and mola slightly crenate, slightly convex without hump and without setae; tuft of setae at apex of mola absent; basal half with dorsally thin setae; incisors with 8 fused denticles. Right mandible ( Figure 11 ): stout prostheca apically maniform; margin between prostheca and mola poorly crenate, without setae, slightly convex; tuft of setae at apex of mola reduced to two small setae; basal half with dorsally short thin setae; incisors with 9 fused denticles. Hypopharynx ( Figure 9 ) with lingua with anteromedial tuft of short robust and superlingua with simple setae. Maxilla ( Figure 12 ): inner margin of the 2 segment of maxillary palp with around 6 hairs and with a shallow subapical excavation leading to a tapering apex; with four denticles on apex of the galealaciniae and 14-16 long setae; maxillary palp extending slightly beyond galealaciniae. Labium ( Figure 13 ) with glossae slender, inner margins with long and stout setae; paraglossae stout, apically flattened, with a row of pectinate setae; the paraglossae more than 1.8 times wider than the glossae. Labial palp 3-segmented; first segment without setae, subequal in length to second and third combined; second segment with a broad thumb-like distomedial projection laterally rounded, third segmentsubconical, inner and outer margins rounded and almost symmetrical, covered with short thin setae and few longer and stouter ones apically near margins.

Thorax: coloration brown without distinct pattern; prothorax brownish; mesothorax and metathorax light brown. Leg: femora with dorsally a row of about 6 pointed setae, rare and long in distal part, smaller and more abundant proximally; ventral margin with numerous acute setae longer apically; without ridged robust setae on dorsomedian surface of femur; tibia dorsally with only few small setae; tarsi with very small setae dorsally; ventral margin with a row of pointed setae slightly increasing in length towards apex; tarsal claw with single row of about 14 teeth progressively increasing in length towards apex ( Figures 6 and 7 ); mid and hind legs similar to foreleg. Hind wing pads absent.

Abdomen: coloration brown without consistent pattern; tergal surface with abundant small scales bases; posterior lateral margins with somewhat regular spines ( Figure 17 ); sterna yellow with scale bases. Gills present on abdominal segments 1–7, well tracheae except first gill with serrations, gill 1 reduced (Figures 15 and 16). Paraproct with numerous marginal spines increasing in length distally and with scale bases scattered over surface; absence of projection at inner distal end ( Figure 14 ). Caudal filaments pale brown to cream with long setae and dark banded appearance at near apex.

### Imagoes

Male imago maximal length of body 5.2 mm ( Figure 18 ); forewing 3.5 mm ( Figure 21 ). Female imago maximal length of body 5.0 mm ( Figure 20 ); forewing 3.5 mm. Hind wing absent in both males and females.

Head: dark brown; turbinate eyes with dark brown tint ( Figure 19 ); ocelli light brown; anterior margin of frons with medial ridge straight in lateral view. Antennae with base of scape and pedicel light brown and flagellum pale yellow. Thorax: dark brown. Forewing hyaline with double intercalary veins between longitudinal 5 crossveins ( Figure 21 ); hind wing absent. Abdomen: coloration of male abdomen, terga reddish brown; red terga (2, 4, 6) alternate with white terga (3, 5); female uniformly brown; terga brown; legs uniformly light yellow.

Male genitalia with three segmented gonopods; first and second segments almost fused, third segment globular; well developed sclerotized process between foreceps, as broad as distance between foreceps, apically flattened without setae ( Figure 22 ).

The larvae and imagoes are associated by rearing.

**Holotype(in alcohol):** Male imago (Ref. No. IE 6); 28.vi.2012. Colls. T. Kubendran, C. Balasubramanian & T. Rathinakumar.

**Paratypes(in alcohol):** 6 male larvae (Ref. No. IE 7), 8 female larvae (Ref. No. IE 8); 28.vi.2012. Coll. T. Kubendran, C. Balasubramanian & T. Rathinakumar. **Holotype** and **paratypes** are deposited at Zoological Survey of India, Southern Regional Center, Chennai, Tamilnadu, India.

### Etymology

The species is named in honour of Dr. T. Soldan for his substantial contribution to the understanding of the Ephemeroptera of Palaearctic and Oriental realms.

### Ecology

The larvae were collected in a small perennial river, Gadana, Tirunelveli District, Tamilnadu, India, latitude (N) 08 ^°^ 47′17.03″, longitude (E) 77 ^°^ 20′49.51″, (2–3 m wide and 0.3 cm depth) with slow water current (0.4 m/sec.) on the eastern part of southern Western Ghats. The water temperature ranged between 22 and 25°C (seasonal variations) and the pH between 6.5 and 7.4. The locality of collection is near the famous Sivasailam temple. The substratum component was mainly gravel with patches of grasses. Organic pollution due to the visit of pilgrims to the temple had a moderate impact on the collection site.

## Discussion

Novikova and Kluge (1987) originally established *Labiobaetis* as a subgenus of *Baetis* Leach (Ephemeroptera: Baetidae) to accommodate a distinct group of species encompassing species of the *Baetis propincuus* group, as defined by Morihara and McCafferty (1979) , distributed in Nearctic, the *B. artibatinus* group, as defined by Müller-Liebenau (1969 , 1973), distributed in Palaearctic, and the *B. molawinensis* group, as defined by Müller-Liebenau (1984) , distributed in Oriental realms. Morihara and McCafferty (1979) showed that these distinct groups of species are defined by unique synapomorphic characteristics not found in other *Baetis* . Thus, they form a monophyletic grouping under *Baetis sensu lato* , with its relative position near the base of the *Baetis* complex, because a phylogenetically significant trait, i.e., the larval villopore is only poorly developed, being rudimentary or absent, and male genital plate is fairly developed. On the other hand, in *Baetis sensu stricto* , the villopore has become well established, but the genital plate has been lost ( McCafferty and Waltz 1995 ).

*Labiobaetis* was raised to the generic rank by McCafferty and Waltz (1995) . Subsequently, Lugo-Ortiz et al. (1999) attempted to consider *Labiobaetis* as a junior synonym of the much-debated genus *Pseudocloeon* Klapalek. However, this synonymy will remain controversial, as pointed out by Gattolliat (2001) , until the larval stage of the type species, *Pseudocloeon kraepelini* Klapalek is known, for which there appears to be a remote possibility in the near future. The genus *Labiobaetis, sensu*Lugo-Ortiz and McCafferty (1997) , fits the Arctogean distributional pattern, i.e., Afrotropical + Holarctic + Oriental regions. However, *Labiobaetis (Pseudocloeon, sensu*Lugo-Ortiz et al. 1999 ) is present in Australia along with three species described by Lugo-Ortiz et al. 1999 . Sixteen species of *Labiobaetis* (three from Sumatra (L. fulmeki * , L. ulmeri * and L. necopinatus*), six from West Malaysia *(L. difficilis, L. diffundus, L. moriharai, L. multus, L. numeratus,* and *L. operates)* one from East Malaysia *(L. borneoensis),* two from Philippines *(L. molawinensis* and *L. sumigarensis),* three from Sri Lanka *(L. geminatus, L. pulchellus,* and *L. ordinatus),* and one from India (L. palmyrae*)) according to placement (tentative*) suggested by McCafferty and Waltz (1995) are so far known from the Oriental realm in addition to the present new species being described from southern Western Ghats of India. In the context of Lugo-Ortiz et al. (1999) having synonymised *Labiobaetis* with *Pseudocloeon* on the basis of adult morphology, as pointed out previously, the species assigned to *Labiobaetis* will find placement in the genus *Pseudocloeon.* However, until the larval and imaginal correspondence is known, the concept of *Pseudocloeon* will remain uncertain. Following a precautionary approach of Gattolliat (2001) , *L. soldani***sp. nov.** along with the above mentioned species from south and southeast Asia will provisionally remain in the genus *Labiobaetis* until global revisions *of Labiobaetis* and *Pseudocloeon* are made.

*Labiobaetis soldani* **sp. nov.** is closely related to *L. pulchellus* ( Müller-Liebenau,1985 ) described from Sri Lanka in the larval stage. However, it can be differentiated from all other species described from the Oriental region by the following combination of characters ( Table 1 ): 1) labrum rounded, with an arc and stout setae, long and thin setae medially; distal margin bordered with setae, distolaterally 14 feathered bristles; 2) inner margin of the 2 segment of maxillary palp with around 6 hairs and with a shallow subapical excavation leading to a tapering apex; 3) the paraglossae of labium more than 1.8 times wider than the glossae; 4) presence of 6 pointed setae on the dorsal femoral margin; 5) absence of hind wing pads; 6) gills in segment 1-7 with serrated margins; and 7) with scattered notched scales in the middle of the paraproct and without a projection on the posterior end of the inner margin of paraproct.

**Table 1:**
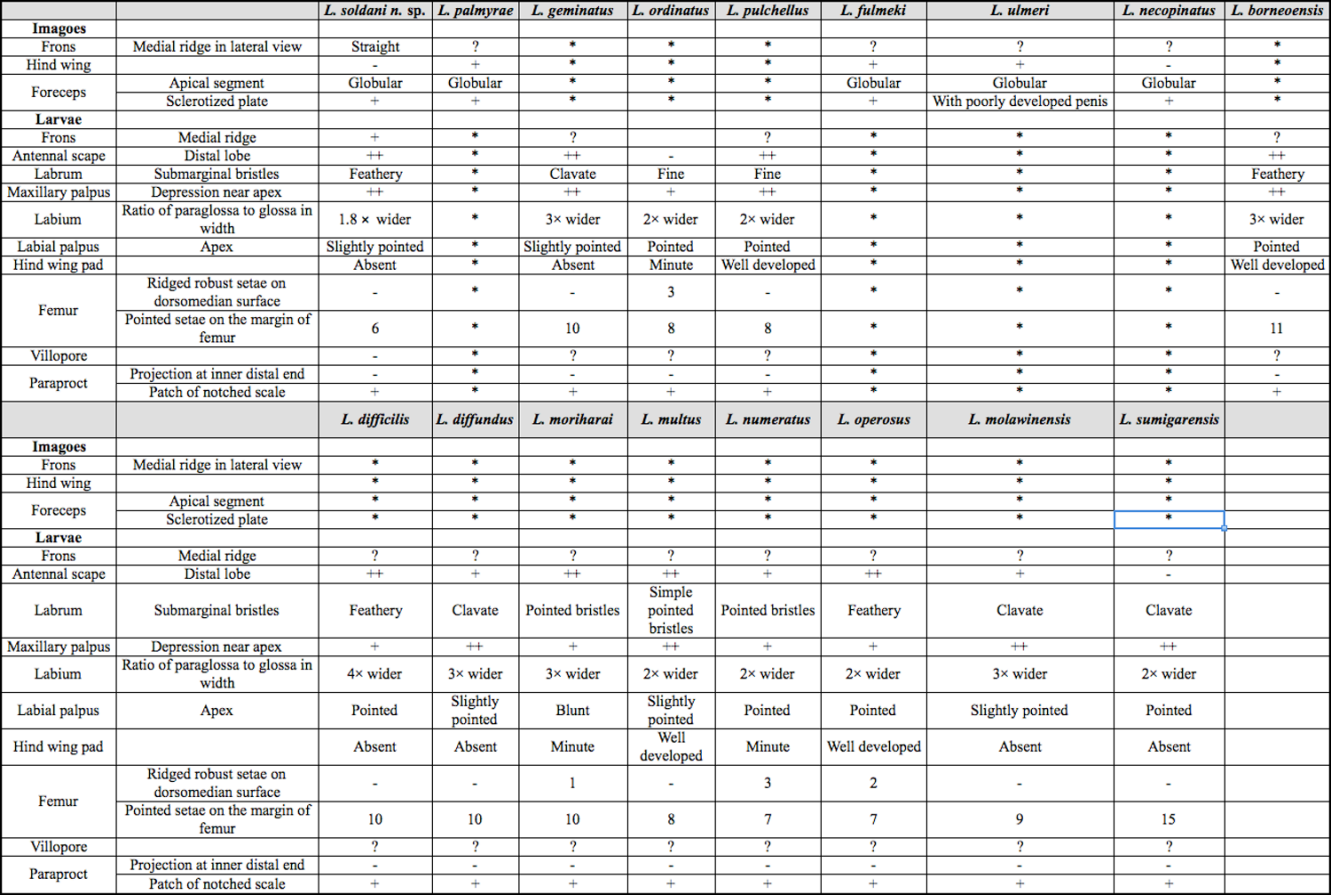
Character states of South and Southeast Asia *Labiobaetis* spp.

(*) Unknown stage (?) Unknown character

Imagoes of *Labiobaetis soldani***sp. nov** are characterized by male genitalia with three segmented gonopods; first and second segments almost fused, third segment globular; well developed sclerotized process between foreceps, as broad as distance between foreceps, apically flattened without setae ( Figure 22 ).
